# Evaluation of the cerebrovascular reactivity in patients with Moyamoya Angiopathy by use of breath-hold fMRI: investigation of voxel-wise hemodynamic delay correction in comparison to [^15^O]water PET

**DOI:** 10.1007/s00234-022-03088-4

**Published:** 2022-11-25

**Authors:** Leonie Zerweck, Till-Karsten Hauser, Constantin Roder, Ganna Blazhenets, Nadia Khan, Ulrike Ernemann, Philipp T. Meyer, Uwe Klose

**Affiliations:** 1 Department of Diagnostic and Interventional Neuroradiology, University Hospital Tuebingen, Hoppe-Seyler-Straße 3, 72076 Tuebingen, Germany; 2 Department of Neurosurgery, University Hospital Tuebingen, Tuebingen, Germany; 3 Department of Nuclear Medicine, Medical Center – University of Freiburg, Faculty of Medicine, University of Freiburg, Freiburg, Germany; 4 Moyamoya Center, University Children’s Hospital Zurich, Zurich, Switzerland

**Keywords:** Cerebrovascular reactivity, Cerebral perfusion reserve capacity, Breath-hold fMRI, [^15^O]water PET, Moyamoya Angiopathy

## Abstract

**Purpose:**

Patients with Moyamoya Angiopathy (MMA) require hemodynamic assessment to evaluate the risk of stroke. Hemodynamic evaluation by use of breath-hold-triggered fMRI (bh-fMRI) was proposed as a readily available alternative to the diagnostic standard [^15^O]water PET. Recent studies suggest voxel-wise hemodynamic delay correction in hypercapnia-triggered fMRI. The aim of this study was to evaluate the effect of delay correction of bh-fMRI in patients with MMA and to compare the results with [^15^O]water PET.

**Methods:**

bh-fMRI data sets of 22 patients with MMA were evaluated without and with voxel-wise delay correction within different shift ranges and compared to the corresponding [^15^O]water PET data sets. The effects were evaluated combined and in subgroups of data sets with most severely impaired CVR (apparent steal phenomenon), data sets with territorial time delay, and data sets with neither steal phenomenon nor delay between vascular territories.

**Results:**

The study revealed a high mean cross-correlation (*r* = 0.79, *p* < 0.001) between bh-fMRI and [^15^O]water PET. The correlation was strongly dependent on the choice of the shift range. Overall, no shift range revealed a significantly improved correlation between bh-fMRI and [^15^O]water PET compared to the correlation without delay correction. Delay correction within shift ranges with positive high high cutoff revealed a lower agreement between bh-fMRI and PET overall and in all subgroups.

**Conclusion:**

Voxel-wise delay correction, in particular with shift ranges with high cutoff, should be used critically as it can lead to false-negative results in regions with impaired CVR and a lower correlation to the diagnostic standard [^15^O]water PET*.*

## Introduction

Moyamoya Angiopathy (MMA) is a progressive occlusive arteriopathy that predominantly affects terminal parts of the internal carotid arteries (ICA) as well as the M1 segment of the middle cerebral artery (MCA) and the proximal anterior cerebral artery (ACA) [1]. The patients typically present with transient ischemic attacks, ischemic or hemorrhagic strokes, headache, or epilepsy [2–4]. The assessment of the cerebral perfusion reserve capacity (CPR) permits the estimation of the risk of ischemic strokes [5]. In case of decreased CPR, neurosurgical revascularization with extracranial-intracranial bypasses is indicated [6, 7]. The estimation of the CPR is performed by functional perfusion imaging [8, 9], whereas [^15^O]water PET with acetazolamide (ACZ) challenge is considered the diagnostic standard [10].

In recent years, the estimation of the cerebrovascular reactivity (CVR) by use of BOLD fMRI gained in importance as alternative imaging method to evaluate the risk of stroke [7, 10–15]. The CVR is defined as the cerebral perfusion change in response to a vasodilatory stimulus, which can be achieved by breath-hold periods (bh) [7, 12, 16–19] or by the inhalation of CO_2_-enriched gas [12, 20–22]. The hypercapnic stimulus is expected to lead to increased cerebral perfusion and thus BOLD signal increase in brain regions with physiological perfusion reserve, whereas no or only a reduced perfusion increase is expected in vascular territories with impaired CVR [23]. In some territories, even BOLD signal decreases, and thus negative CVR values may be observed [21, 23, 24]. This phenomenon might be explained by adjacent vascular territories that show increased perfusion during hypercapnic stimulation at the expense of regions with lower CVR and considered as a so-called steal phenomenon [21, 23, 24]. Advantages of hypercapnia-triggered BOLD fMRI over [^15^O]water PET include higher availability and lower cost of the procedure, since no short-lived radiopharmaceuticals are required [11]. bh-fMRI necessitates patients’ cooperation [11] but does not require MR-compatible gas delivery equipment, uncomfortable facemasks [25, 26], and complex monitoring during CO_2_ administration [27]. Thus, bh-fMRI is broadly available and easily implementable [11]. Hemodynamic evaluation of patients with MMA by use of bh-fMRI has been suggested as a comparable alternative to the diagnostic standard [^15^O]water PET [7] with high reproducibility [19].

Recent studies recommend voxel-wise correction of regional hemodynamic-related delays in the signal time-courses when assessing the CVR by use of hypercapnia-triggered fMRI [28, 29]. The hemodynamic response to vasodilative stimuli might show different temporal dynamics in different brain regions, both in patients with cerebrovascular diseases and in healthy individuals [28]. The underlying cause of temporally delayed BOLD signals might be regionally different blood arrival times, as well as regionally time-delayed cerebral vessel responses [29]. Especially in patients with MMA, hemodynamic time lags resulting from cerebral perfusion via collateral vessels might be relevant [30]. In previous studies concerning hypercapnia-triggered fMRI, higher CVR values were described after voxel-wise time delay correction [31, 32].

To our best knowledge until now, the effect of a time delay correction on the estimation of CVR by use of bh-fMRI has not been investigated in a large number of patients with MMA. In particular, it has not yet been assessed whether higher CVR values after time delay correction also lead to a more precise estimation of the cerebral perfusion reserve, as the estimation of the CVR with time delay correction has not yet been compared to an established reference method such as [^15^O]water PET. Another unanswered question is whether voxel-wise time delay correction might lead to an overestimation of the CVR in territories with “vascular steal phenomenon” and previously negative or low CVR values.

The aim of this study was to investigate the effect of voxel-wise hemodynamic time delay correction on the estimation of the CVR by use of bh-fMRI in patients with MMA and to compare the results to the assessment of the CPR yielded by the diagnostic standard [^15^O]water PET with ACZ challenge.

## Methods

### Participants

A retrospective analysis of bh-fMRI data sets and the corresponding [^15^O]water PET data sets of 22 patients with MMA was performed. Both examinations were performed as routine clinical scans. At our Moyamoya Center, we usually perform an initial [^15^O]water PET examination and the first bh-fMRI examination within 1–3 months. Afterwards we regularly perform bh-fMRI follow-up examinations as they are broadly available, less expensive, and do not require radioactive tracers. If a bh-fMRI examination reveals a decreased CVR, we perform a complementary [^15^O]water PET examination before indicating neurosurgical revascularization. For this reason, we acquired bh-fMRI data sets and [^15^O]water PET data sets for answering the same clinical question.

Inclusion criteria of the study were angiographically proven MMA and the availability of MRI and [^15^O]water PET examinations at the most 4 months apart with no revascularization therapy in between. Exclusion criteria were secondary cerebral diseases. General patient data can be found in Table 1. Ethical approval was obtained at the Local Ethics Committee. All patients included in this study signed a general agreement prior to their hospital stay allowing processing, archiving, and publication of all patient data.

**Table 1:** Overview of general patient data

General patient data	
Mean age (range)	45 (10–67)
Female/male	16/8
Bilateral Moyamoya disease	18
Unilateral Moyamoya Angiopathy	4
bh-fMRI data sets	22
[^15^O]water PET data sets	22
MRI data sets with steal phenomenon	7
MRI data sets with territorial time delay	9
MRI data sets with neither steal phenomenon nor territorial time delay	6
MRI data sets without steal phenomenon and territorial time delay	6

### MRI data acquisition

The MR images were obtained on a 3-T MR scanner (Magnetom Skyra, Siemens, Erlangen, Germany). During the examination, patients were placed in supine position on the scanner table. All data were acquired using a standard 20-channel head coil. The fMRI data acquisition was performed by means of T2*-weighted echo-planar sequences with the following parameters: TR = 3000 ms, TE = 36 ms, matrix 96 × 96, 3-mm slice thickness, 34 slices in interleaved ascending order, FOV = 245 mm, resolution 2.6 × 2.6 × 3.0, echo spacing: 0.58 ms, TA 6:53 min, and 135 measurements.

The breath-hold paradigm included 60 s of normal breathing, followed by 5 repetitive cycles, each involving 9 s end-expiratory breath-hold periods and 60 s of regular breathing. The breathing instructions were visually delivered via a wall-mounted display by use of a mirror affixed to the patient’s head coil. Presentation V20.1 (Neurobehavioral Systems, Berkeley, CA, USA) was applied to present the scanner triggered stimuli. Before the bh-fMRI examination, the breath-hold paradigm was explained to the patients, and the patients practiced the procedure outside the scanner.

### MRI data preprocessing

**Fig. 1:**
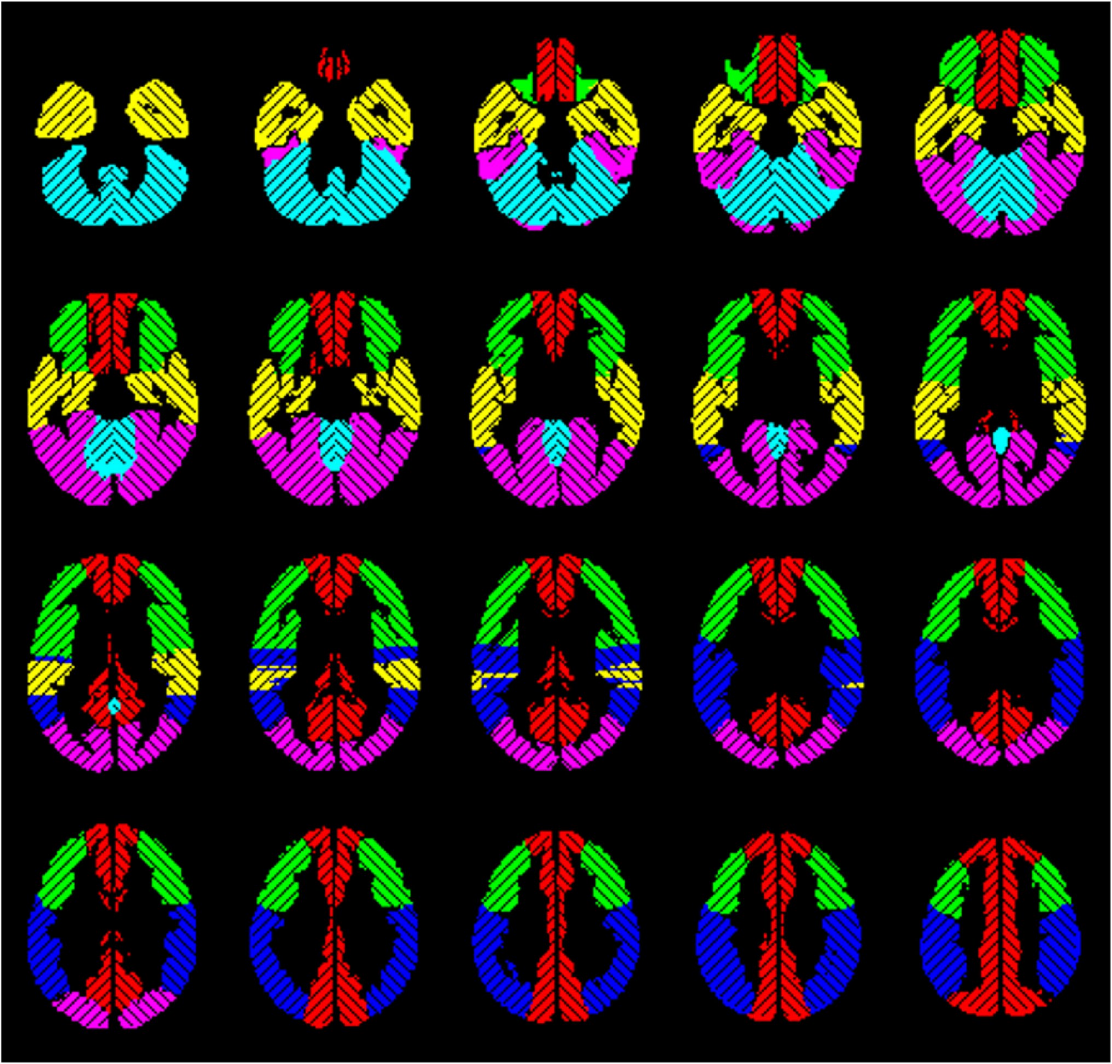
Volumes of interest based on the vascular territories of the anterior cerebral artery (red), the middle cerebral artery (frontal (green), temporal (yellow), and parietal (blue)), the posterior cerebral artery (pink), and the cerebellum (turquoise) separated by side [17]

The MRI data preprocessing was performed using Statistical Parameter Mapping (SPM12) (https://www.fil.ion.ucl.ac.uk/spm/) running on MATLAB (R2018b (The MathWorks, Inc., Natick, Massachusetts; http://www.mathworks.com)). The DICOM images were converted to analyze format in NIfTI (Neuroimaging Informatics Technology Initiative), slice-timing corrected to equalize the time of image acquisition, realigned to correct subjects’ head movement, normalized to standard MNI space, spatially smoothed by a Gaussian kernel of 12-mm FWHM, and temporally interpolated by a factor of 4, resulting in a temporal resolution of 0.75 s. The mean CVR within vascular territories was analyzed by using a template in MNI space with 12 predefined volumes of interest (VOI) based on the arterial transit time flow territories [33, 34] (see Fig. 1). All further data processing was performed by use of in-house scripts programmed in MATLAB.

### Analysis of bh-fMRI data

The first step was to verify patients’ compliance in performing the breath-hold paradigm. We used an approach proposed by Hauser et al. [7] and recently applied [17, 19]: we utilized the above-mentioned predefined VOI of the cerebellum [33, 34] (see Fig. 1) to calculate the mean cerebellar signal time-course. The cerebellum is supplied by vertebrobasilar arteries that are unaffected by MMD and represents a region where a physiological hemodynamic response is expected [7, 17, 18, 35]. If the cerebellar signal time-course revealed no expected BOLD signal peak in visual inspection, we assumed a lack of compliance and excluded the bh-fMRI data set from the further analysis.

Then, the bh-fMRI data sets were evaluated with and without voxel-wise time delay correction:

1. Analysis of the CVR without time delay correction
  The signal time-courses of each voxel were averaged over the five cycles of the respiratory paradigm. Then, the cross-correlation between each voxel’s signal time-course and the mean signal time-course of the cerebellum was calculated, and the cross-correlation coefficients were used as parameters of the CVR.
2. Analysis of the CVR with time delay correction
  The mean cerebellar BOLD signal time-course was determined. Subsequently, time-shifted signal time-courses of each voxel relative to the mean cerebellar signal time-course were calculated. For this purpose, the signal time-course of each voxel was time shifted up to 15 s to the left and up to 30 s to the right. Due to the temporal resolution of 0.75 s, 20 negative and 40 positive time shifts resulted.
  The next step was to calculate the cross-correlation between each time-shifted time-course and the cerebellar signal time-course. Therefore, the mean cerebellar signal time-course was averaged over the five cycles of the respiratory paradigm. The time shifting of the signal time-course of each voxel resulted in a smaller number of overlapping measurement points. For this reason, averaging each voxel’s time-shifted signal time-course over all five cycles was no longer possible, and the time-shifted signal time-courses were averaged over only four cycles. After that, analogous to (1), the cross-correlation between the mean signal time-course of each voxel and the mean signal time-course of the cerebellum was calculated.
  Due to different localization of the voxels, individually different hemodynamic-related delays of the signal response were expected. Therefore, when determining the CVR, the aim was not to apply the same time shift to each voxel’s signal time-course but to apply individual time shifts within certain predefined time shift ranges. For this reason, we examined different time shift ranges and, within these time shift ranges, used the maximum correlation coefficient of the signal time-course of each voxel and the cerebellar signal time-course as parameter of CVR. We varied the low cutoff (− 15 to 0 s in steps of 0.75 s) and high cutoff (0 to 30 s in steps of 0.75 s) of the time shift ranges. As we combined each low cutoff with each high cutoff, 21 × 41 = 861 different shift ranges resulted. For example, if a time shift range with low cutoff =  − 3 s and high cutoff = 9 s was examined, it was possible that one voxel reached its maximum cross-correlation value when its signal time-course was shifted − 2 s relative to the cerebellar time-course, while another voxel reached its maximum after time shifting of + 6 s.

### [^15^O]water PET with ACZ challenge: data acquisition, processing, and analysis

PET data acquisitions and analyses were performed as previously described using a semi-quantitative approach to avoid the need for arterial blood sampling [7, 17]. In brief, after a low-dose CT for attenuation correction, four 4-min scans at an inter-scan interval of 10 min were acquired per patient on a Philips Gemini TF64 PET/CT (*n* = 2; until 12/2017) or a Philips Vereos digital PET/CT system (*n* = 20; from 01/2018 onward; Philips, The Netherlands) after intravenous slow-bolus (3 s) injection of 300 MBq [^15^O]water (10-ml volume). Two scans were done before, and two scans at 5 and 15 min after a 5-min infusion of ACZ (1000 mg in 10 ml; *n* = 2 received 800 mg because of low body weight of 53 and 55 kg). PET data sets were reconstructed into a dynamic sequence of 30 frames (18 × 5 s, 9 × 10 s, and 3 × 20 s) using a vendor-specific 3D iterative reconstruction. For simplified voxel-wise calculation of CPR maps, all scans of each subject were corrected for possible head motions and integrated over 60 s after arrival of the tracer in the individual’s brain (according to a whole-brain time-activity curve). After averaging the two integral scans before and after ACZ administration and applying an empirical whole-brain segmentation (mask threshold: 18% of image maximum), voxel-wise estimates of CPR were created by calculating the signal change (%) in each voxel from baseline to ACZ stimulation. These parametric images were smoothed (Gaussian filter, 12-mm FWHM) and masked (see above) to give the final CPR maps. We used a customized “UCLA 2” color scale for display of the CPR maps. For VOI analyses (see MRI data preprocessing), CPR maps were spatially normalized using a PET template in MNI space (provided in Statistical Parametric Mapping (SPM 12, http://www.fil.ion.ucl.ac.uk/spm/)) and baseline integral images for estimation of individual normalization parameters. The commercial software packages PMOD (version 3.7; PMOD Technologies LLC, Switzerland) and MATLAB (The MathWorks, Inc., Natick, MA, USA), as well as the freely available SPM 12 were used for the aforementioned analyses.

### Comparative analysis of bh-fMRI and [^15^O]water PET

The mean CVR and CPR values (bh-fMRI: correlation coefficients between the signal time-courses of each voxel and the mean cerebellar signal time-courses, [^15^O]water PET: approximate blood flow changes (%) after ACZ stimulation of each voxel) of the above-mentioned 12 VOIs of each patient were calculated. We calculated the correlation between the bh-fMRI CVR values and the [^15^O]water PET CPR values with Pearson’s correlation coefficient *r*, comparing the mean CVR/CPR of the 12 VOIs.

We investigated the effect of voxel-wise time delay correction of patient-specific and standardized shift ranges comparing the correlation between bh-fMRI and [^15^O]water PET before and after time delay correction within different shift ranges by use of paired sample *t* tests.

**Fig. 2:**
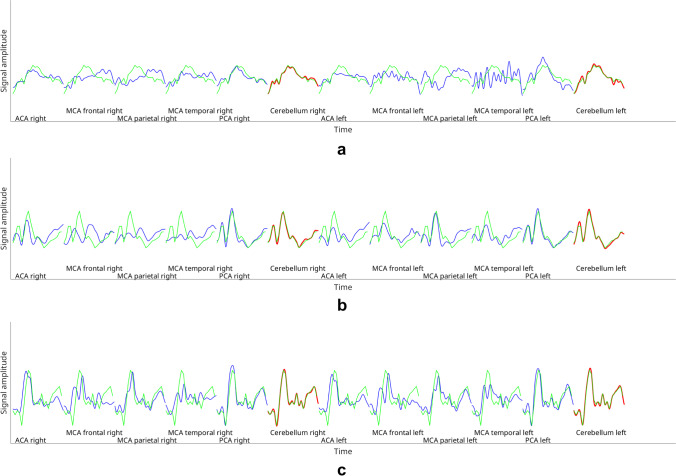
Exemplary averaged bh-fMRI BOLD signal time-courses of each VOI of **a** one data set with steal phenomenon (visible in the territory of the left parietal MCA), **b** one data set with territorial time delay (visible in the territories of the right frontal and parietal MCA), and **c** one data set with neither steal phenomenon nor territorial time delay. The cerebellar signal time-courses are shown in red and the other VOIs’ signal time-courses in blue. Superimposed on each signal time-course is the mean cerebellar signal time-course in green. The duration of each averaged cycle was 69 s

The effect of time delay correction was examined for the entire cohort, as well as in subgroups. We therefore divided the bh-fMRI data sets into three subgroups based on their BOLD signal time-courses without time shift correction: (1) data sets showing negative CVR-values, indicating inverse signal response (so-called steal phenomenon) in at least one VOI, (2) data sets with territorial time delay of at least 3 s relative to the cerebellum in at least one VOI, and (3) data sets with neither steal phenomenon nor territorial time delay between the evaluated VOIs (see Fig. 2).

We calculated the mean intra-subject correlation between the individual bh-fMRI and the [^15^O]water PET data sets. For each test, uncorrected *p* < 0.05 was considered statistically significant.

## Results

In total, 24 bh-fMRI data sets and the corresponding [^15^O]water PET data sets acquired between 2016 and 2021 were analyzed and compared. Visual inspection of the cerebellar signal time-courses revealed that the respiratory instructions were followed correctly by 22 patients. The data sets of two patients showed no signal adequate peaks in the cerebellar reference regions indicating inadequate compliance of the patients and were therefore excluded from further analysis.

### Exemplary data evaluation

**Fig. 3:**
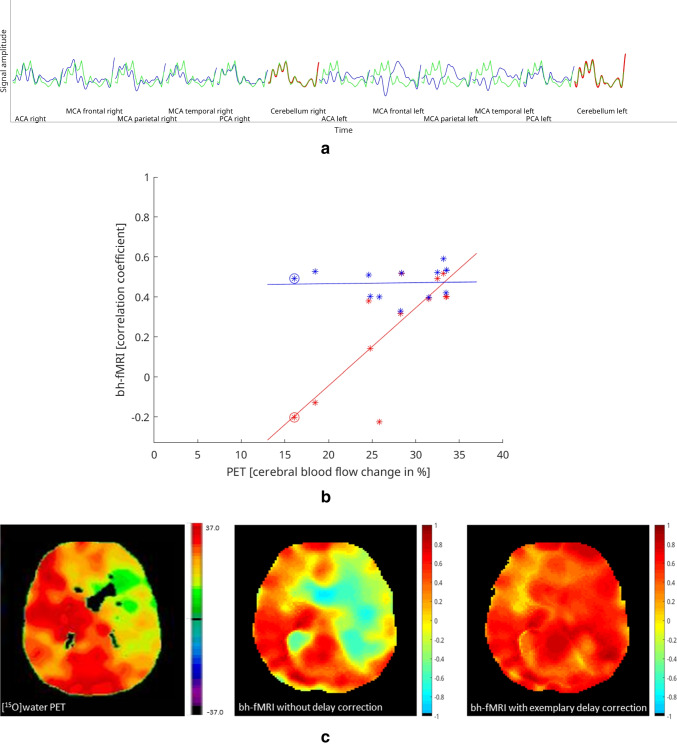
Exemplary results without and with time delay correction of one moyamoya patient. **a** Mean signal time-courses of each VOI without time delay correction. The cerebellar signal time-courses are shown in red and the other VOIs’ signal time-courses in blue. Superimposed on each signal time-course is the mean cerebellar signal time-course in green. **b** A scatter plot indicating the correlation between bh-fMRI and [^15^O]water PET without time delay correction (red, *r* = 0.81) and with exemplary time delay correction within the shift range 0–20 s (blue, *r* = 0.02). **c** Corresponding [^15^O]water PET map and the bh-fMRI maps without time delay correction and after time delay correction within the shift range 0–20 s**.** Both the scatterplots and the maps reveal a significantly weaker correlation between bh-fMRI and [^15^O]water PET after performing the delay correction than without delay correction

An exemplary evaluation with voxel-wise time delay correction of one single data set is shown in Fig. 3. Figure 3a presents the mean signal time-courses of each VOI. In the VOIs of the frontal, temporal, and parietal right MCA, territorial time-delayed BOLD signal increases relative to the cerebellar signal increase can be seen. Consequently, the CVR values in these VOIs were low (see, for example, the circled red mark in Fig. 3b), leading to a high correlation between bh-fMRI and [^15^O]water PET, which reveals low CPR values in the respective VOIs. For the exemplary data set, the bh-fMRI data analysis with delay correction within the shift range 0–20 s was performed. As seen in Fig. 3b, the delay correction resulted in higher CVR values overall, especially in the VOI corresponding to the circled blue mark with previously low CVR values. This resulted in a weaker correlation between bh-fMRI and [^15^O]water PET (*r* = 0.02 vs. 0.81). The exemplary maps in Fig. 3c reveal a higher agreement between bh-fMRI and [^15^O]water PET when processing the bh-fMRI data without time delay correction. After applying the time delay correction within the exemplary shift range, regions with previously low CVR show higher CVR values. This overestimation results in lower similarity with the [^15^O]water PET data sets.

### Correlation between bh-fMRI and [^15^O]water PET without time delay correction

**Fig. 4:**
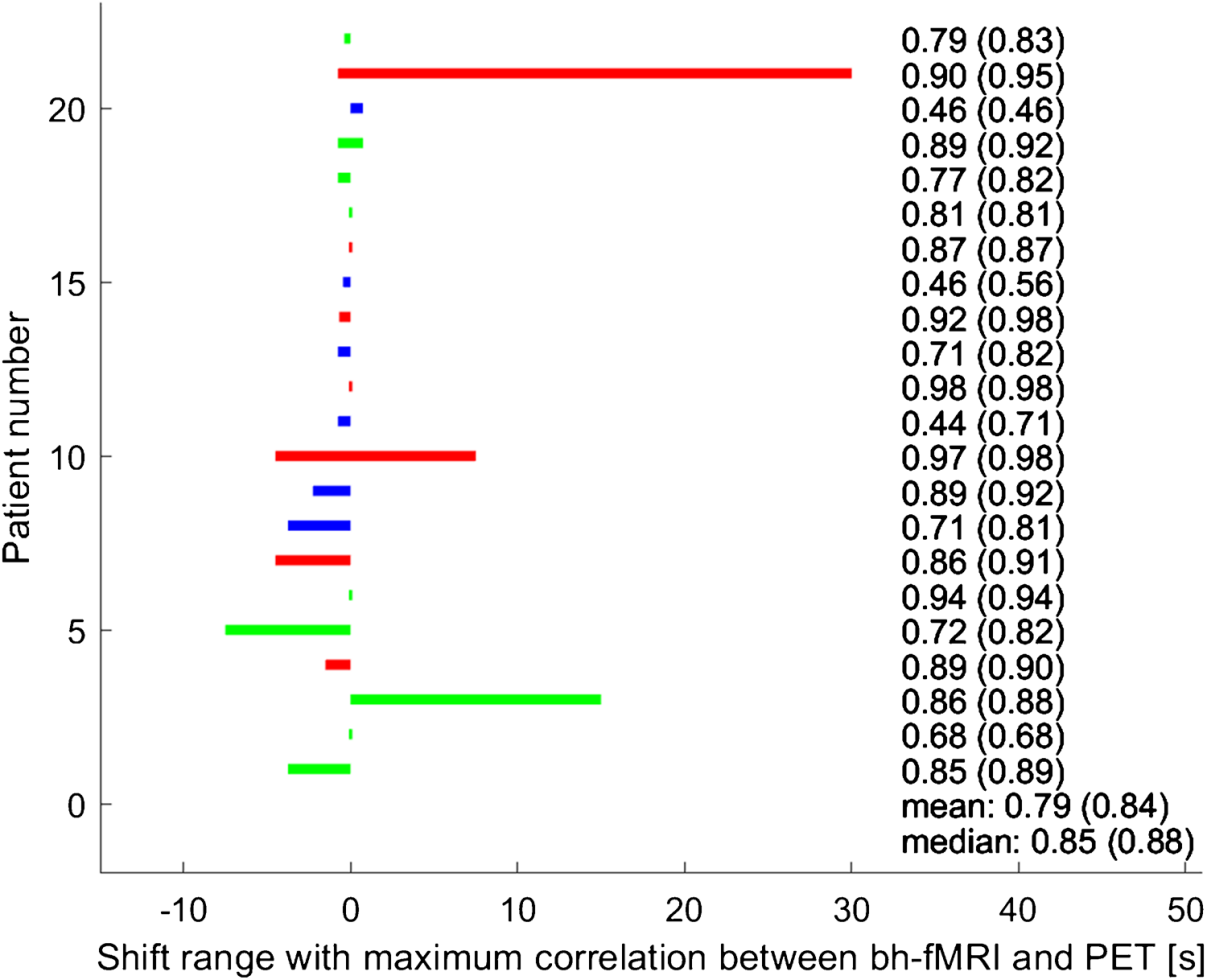
Patient-specific shift ranges with maximum correlation between the bh-fMRI data set and the corresponding [^15^O]water PET data set. The two right columns indicate the correlation between bh-fMRI and [^15^O]water PET without and with delay correction for each patient. The values in parentheses correspond to the correlation after the delay correction that is shown by the colored bars. Red bars indicate data sets with steal phenomenon, green bars indicate data sets with territorial delay, and blue bars represent data sets with neither steal phenomenon nor territorial delay. All correlations were significant at *p* < 0.05

In all patients, there was a significant correlation between the bh-fMRI and the [^15^O]water PET data sets (see Fig. 4). The mean correlation between the bh-fMRI and the [^15^O]water PET data sets was 0.79 ± 0.16 (0.44–0.98, *p* < 0.001).

### Voxel-wise time delay correction with patient-specific shift range

Applying voxel-wise time delay correction within patient-specific shift ranges with maximum correlation between bh-fMRI and [^15^O]water PET resulted in a significant higher agreement between bh-fMRI and [^15^O]water PET (*r* = 0.84 vs. 0.79, *p* < 0.01) (see Fig. 4). As the mean results were markedly influenced by the results of one patient (patient 11) who showed a distinctly higher correlation after delay correction (*r* = 0.71 vs. 0.44), we additionally calculated the median correlation between bh-fMRI and PET before and after delay correction. In this case, a slightly improved agreement between bh-fMRI and [^15^O]water PET was also seen (median = 0.85 vs. 0.88) (see Fig. 4). However, it is noticeable that the shift ranges with maximum correlation between bh-fMRI and [^15^O]water PET differ for all patients. For this reason, in a next step, the group-averaged correlation between bh-fMRI and [^15^O]water PET within standardized and identical shift ranges for all patients was calculated.

### Voxel-wise time delay correction with standardized shift range

**Fig. 5:**
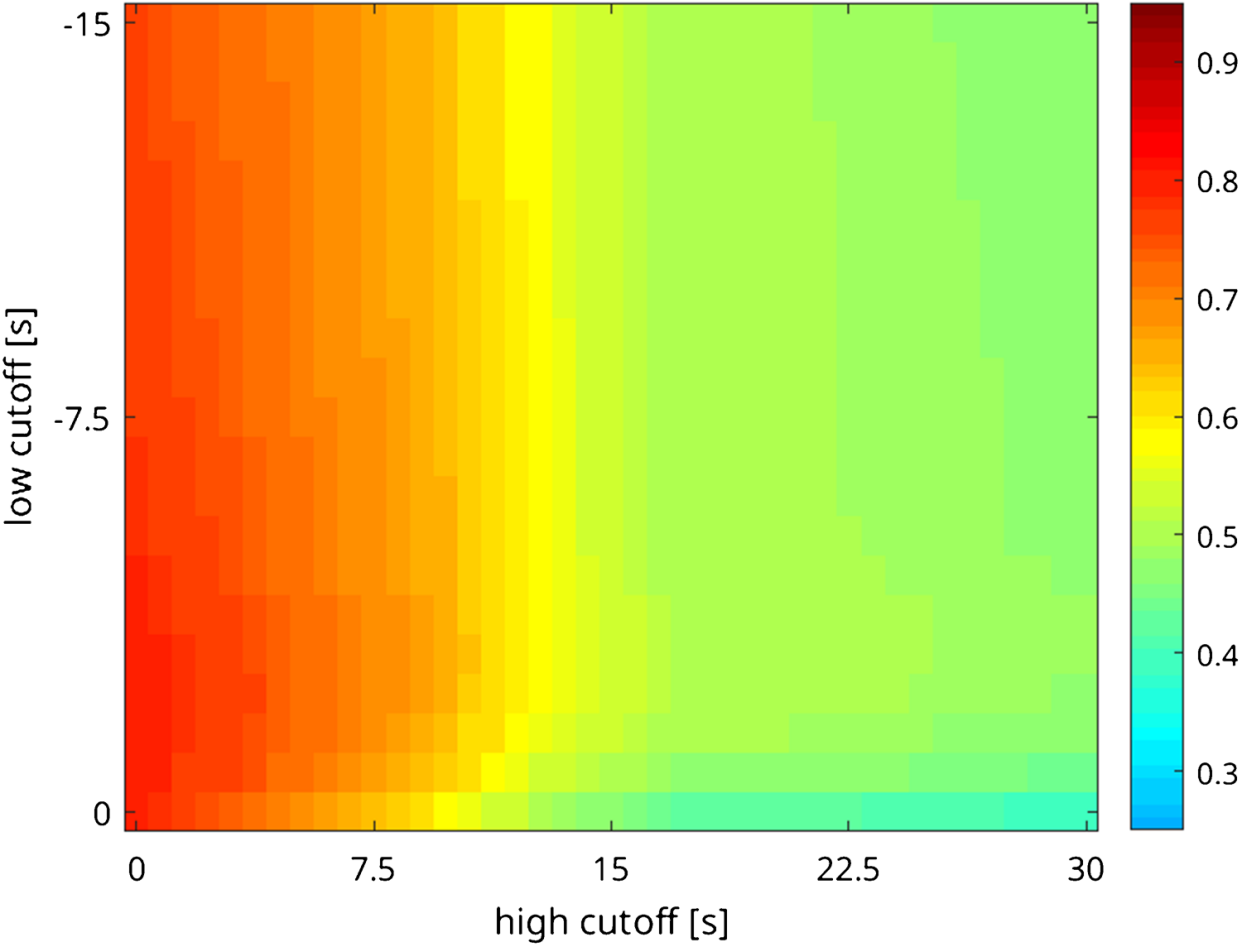
Group-averaged correlation between bh-fMRI and [^15^O]water PET evaluating different shift ranges with variable low cutoff and high cutoff. The mean correlation between bh-fMRI and [^15^O]water PET is maximal at a standardized shift range with low cutoff =  − 2.25 s and high cutoff = 0 s, but not significantly higher than the correlation without delay correction (*r* = 0.81 vs. 0.79, *p* = 0.15)

Figure 5 shows the group-averaged correlation between bh-fMRI and [^15^O]water PET evaluating different standardized shift ranges with variable low-cutoff and high-cutoff shift range. The shift range with low cutoff =  − 2.25 s and high cutoff = 0 s resulted in the highest correlation between bh-fMRI and [^15^O]water PET (*r* = 0.79 vs. 0.81), whereas the improvement was slight and not significant (*p* = 0.15). Distinctly lower correlations were observed when shift ranges with positive high cutoff were applied.

**Fig. 6:**
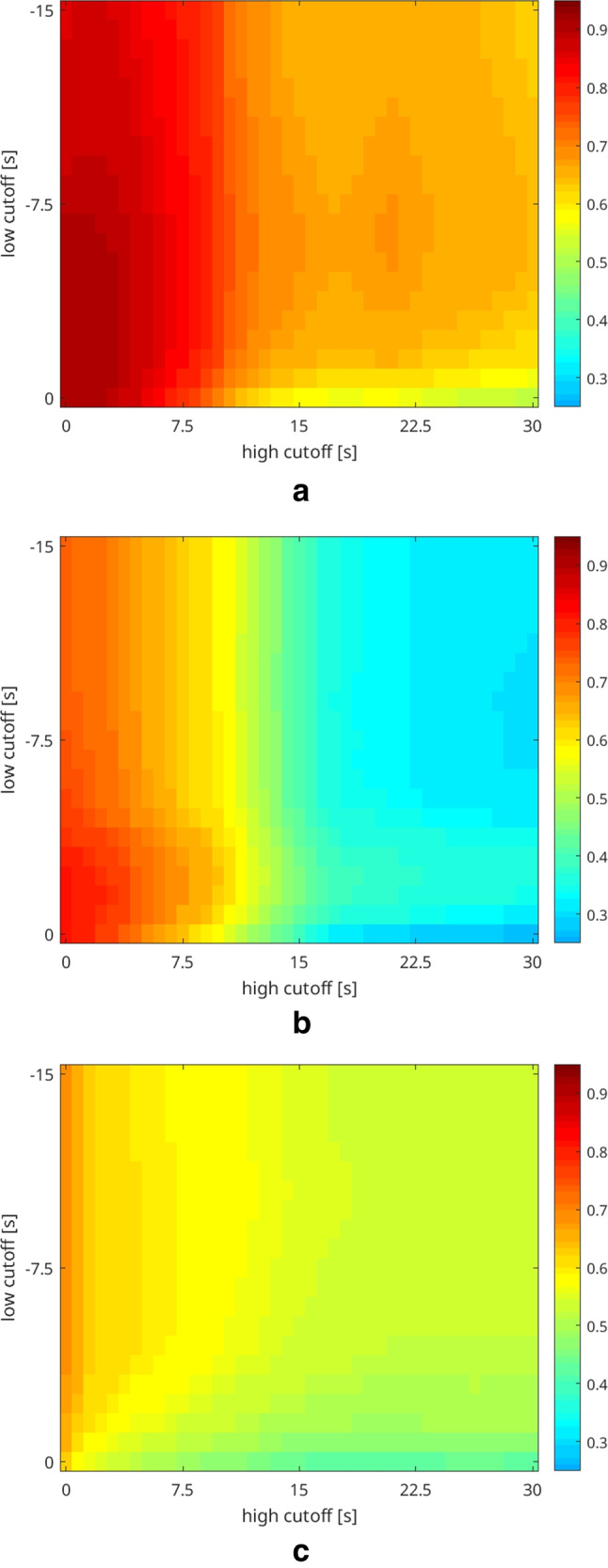
Mean correlation between bh-fMRI and [^15^O]water PET evaluating different shift ranges with variable low cutoff and high cutoff. The patients are sorted by subgroup affiliation: **a** steal phenomenon, **b** territorial time delay, and **c** neither steal phenomenon nor territorial time delay. Depending on the subgroup, the following shift ranges optima result: steal phenomenon, shift range − 1.50–0 s; territorial delay, shift range − 0.75–0 s; and neither steal phenomenon nor territorial delay, shift range − 11.25–0 s. No subgroup revealed a significant improvement in the correlation between bh-fMRI and [^15^O]water PET

The results of the subgroup analysis based on the signal time-courses (steal phenomenon, territorial time delay, and neither steal phenomenon nor territorial time delay) are shown in Fig. 6. In the subgroups with steal phenomenon and territorial time delay, the correlation between bh-fMRI and [^15^O]water PET became maximal when an analysis of the bh-fMRI data sets with minimal delay correction was performed (Fig. 6a, b). However, in both subgroups, the improvement in the correlation between bh-fMRI and [^15^O]water PET was slight and not significant at a sample size of seven and nine patients. In the subgroup with steal phenomenon, the agreement between bh-fMRI and [^15^O]water PET was maximal after time delay correction with low cutoff =  − 1.50 s and high cutoff = 0 s (*r* = 0.92 vs. 0.90, *p* = 0.59). In the subgroup with territorial time delay, the correlation was maximized after delay correction with low cutoff =  − 0.75 s and high cutoff = 0 s (*r* = 0.82 vs. 0.81, *p* = 0.28). The subgroup with neither steal phenomenon nor territorial time delay revealed the highest correlation between bh-fMRI and [^15^O]water PET after application of a time shift correction with low cutoff =  − 11.25 s and high cutoff = 0 s (*r* = 0.68 vs. 0.63), but with a sample size of six patients, the increase in the correlation was not significant (*p* = 0.35) (Fig. 6c).

## Discussion

Estimation of CVR is considered a biomarker of cerebrovascular health [28, 29, 31]. An important scope of application is the cerebrovascular moyamoya disease [7, 17–19, 29, 36]. A previous study suggests bh-fMRI as a broadly available and less expensive alternative to the diagnostic standard [^15^O]water PET in patients with MMA [7]. bh-fMRI is characterized by its simple feasibility, which requires only little additional equipment and can be performed on standard clinical scanners [25, 26]. The breath-hold periods lead to an arterial CO_2_ increase, which causes the cerebral vasodilatation. The change in perfusion leads to a measurable BOLD signal increase through a relative decrease of deoxyhemoglobin [29]. Previous studies suggested that local temporal delays in the hemodynamic response may lead to a false-low estimation of the CVR [31] and recommended a voxel-wise delay correction [28]. It has been described that voxel-wise delay correction can lead to higher CVR values [31, 32].

The aim of this study was to investigate voxel-wise time delay correction in patients with MMA. To evaluate the impact of hemodynamic delay correction, we compared the data sets after time shift correction within different shift ranges with variable low- and high-cutoff value with [^15^O]water PET. To our knowledge, bh-fMRI was compared to the diagnostic standard [^15^O]water PET in patients with MMA in a visual and semi-quantitative analysis in only one study to date [7]. A quantitative comparison between bh-fMRI and [^15^O]water PET in respect to time delay was not yet performed.

Even in individuals without cerebrovascular diseases, regional hemodynamic-related differences in the BOLD signal response time are expected [31]. For example, earlier signal responses were observed in the gray matter compared to the white matter [31]. The cerebellum was described as a region with late BOLD signal response [31]. In this study, we used the cerebellar signal time-course as reference time-course for the cross-correlation analysis. For this reason, we investigated time shifts in both directions relative to the cerebellum, although in patients with MMA, in territories supplied via collateral vessels a delayed signal response is more likely than a too early signal response.

In this study, we demonstrated a better agreement between bh-fMRI and the diagnostic standard [^15^O]water PET when delay correction with patient-specific shift ranges was applied. However, in clinical diagnostics, it is obviously not constructive or beneficial to determine the optimal shift range with knowledge of the PET data sets and afterwards apply it to the bh-fMRI data sets. Rather, bh-fMRI should be usable without knowledge of the PET data sets as an alternative, not supplement to [^15^O]water PET. For this reason, we investigated whether a shift range exists in which a higher agreement between the bh-fMRI and the [^15^O]water PET data sets can be observed in all patients and is therefore suitable for clinical application where no PET data is available.

Our analysis of 22 data sets revealed no significantly improved mean correlation between bh-fMRI and [^15^O]water PET after time delay correction with any shift range. The highest correlation was found after voxel-wise time delay correction within the shift range − 2.25–0 s, although the improvement was slight and non-significant. For other shift ranges, especially with high cutoff, a distinctly lower agreement between bh-fMRI and [^15^O]water PET was found. The subgroup analysis (data sets with steal phenomenon, territorial delay, neither steal phenomenon nor territorial delay) also revealed a negligible and non-significant improvement in the correlation between bh-fMRI and the diagnostic gold standard [^15^O]water PET, especially in severely pathologic data sets where steal phenomena or territorial time delays in the signal time-courses were observed. Only in data sets with neither steal phenomena nor territorial time delays a slightly improved correlation between bh-fMRI and [^15^O]water PET was measurable after standardized shift ranges. The weaker agreement between bh-fMRI and [^15^O]water PET after time delay correction within most of the shift ranges may result from the disproportionate increased CVR in regions with previously lower CVR, in particular in regions with steal phenomenon or territorial delay. This may be due to the false identification of the peak of the response curve. Especially in areas that show marked reduction of signal increase, a so-called recovery peak is sometimes visible which can be falsely identified as the stimulus response. This would dramatically increase the estimated CVR in this area and lead to false-negative results. After delay correction, the bh-fMRI data sets correlate worse with the diagnostic standard [^15^O]water PET, in which the affected regions show low CPR values. This could be one reason why the correlation between bh-fMRI and [^15^O]water PET decreases markedly after delay correction with positive high cutoff.

The slightly improved correlation after delay correction with modified low cutoff might be explained by the adjustment of the delayed cerebellar BOLD signal response as described above and previously reported [31]. Shift ranges with negative low cutoff could lead to constantly low CVR values in regions affected by MMD but higher CVR values in healthy regions with previously false-positive low CVR values due to delayed signal responses. However, as already mentioned, these effects are small.

In previous studies regarding hypercapnia-triggered fMRI, time delays of a few seconds were described [31, 37, 38]. We intentionally did not preselect the time shift ranges by evaluating distinctly larger time shift ranges than physiologically plausible. Since perfusion delays are most likely to be expected in stenosing vascular disease, we evaluated larger high cutoffs than low cutoffs. It should be considered that the voxel-wise delay correction within large time shift ranges might lead to a coincidental similarity between the signal time-courses, particularly in time periods with high noise. This could result in an overestimation of the CVR values and lead to a minimization of regional differences in the CVR and to false-negative results in the affected regions.

Despite the increasing relevance of the bh-fMRI approach [28], no standardized procedure for processing the raw data exists so far [28]. In addition to bh-fMRI, there exist other hypercapnia-triggered fMRI techniques, such as the inhalation of CO_2_-enriched gas or rebreathing [28]. Recent studies also suggest resting-state fMRI (rs-fMRI), where no CO_2_-enriched gas administration or respiratory paradigms take place [17, 27, 28, 36]. The method relies on CO_2_ alterations due to intrinsic respiratory variations during a task-free resting-state scan [17, 27, 28, 36]. Some fMRI studies recommend to evaluate the magnitude of the signal change and the temporal component of the signal change of the hypercapnia-triggered fMRI data separately [29]. The results of our study suggest that a combination of the parameters in the estimation of CVR may lead to a lower agreement with the diagnostic standard [^15^O]water PET. The optimal time-shift range observed in this study that reveals a slightly improved correlation between bh-fMRI and [^15^O]water PET is not exactly transferable to other studies because of different applications of hypercapnia-triggered fMRI (CO_2_-enriched gas inhalation, rebreathing, bh-fMRI with different breath-hold durations, rs-fMRI). However, the observation that fMRI time delay corrections can lead to a weaker correlation to the diagnostic standard [^15^O]water PET is highly relevant for further improving the evaluation of different hypercapnia-triggered fMRI approaches. Further studies are needed to better understand the physiological background and to continue to methodologically optimize the procedure.

This study confirms the results of Hauser et al. [7], demonstrating a high correlation between bh-fMRI and the diagnostic standard [^15^O]water PET. A limitation of bh-fMRI remains the need and assurance of patients’ cooperation. In this retrospective study, we used an approach proposed by Hauser et al. and verified patients’ compliance by evaluating the cerebellar signal time-courses [7]. Another possibility to verify the compliance is monitoring patients’ respiratory movements during the bh-fMRI examination by means of a pneumatic abdominal belt [18]. Due to the confirmed high agreement to [^15^O]water PET and the broad availability, we consider bh-fMRI, despite the disadvantage of the necessary patients’ cooperation and only a semi-quantitative hemodynamic evaluation, as a helpful method, which is already applied in clinical routine for follow-up examinations [19].

## Conclusions

Breath-hold fMRI is a promising method to estimate the CVR in patients with MMA as it reveals a high agreement with the diagnostic standard [^15^O]water PET. A voxel-wise time delay correction should be critically evaluated, as worse or only minimally better correlations between [^15^O]water PET and bh-fMRI can be measured, especially for large time shift ranges.

## Data Availability

The data that support the findings of this study are available from the corresponding author, upon reasonable request.
